# Supplementary material for: Early life events in functional abdominal pain disorders in children
Source: PLoS One. 2022 Nov 2;17(11):e0275419. doi: 10.1371/journal.pone.0275419 (PMC9629606; doi:10.1371/journal.pone.0275419)
Supplement: S1 Questionnaires — (PDF) [file pone.0275419.s001.pdf]

The study of functional abdominal pain among 5-12 years old children

No ………………..

Date …………………………………..

1. Name……………………………………………………….
2. Age…………………………………………………………

3. Gender Male/Female Male [ ] Female [ ]

4. Address………………

5. Contact number……………………………………………….

6. Dominant hand Right [ ] Left [ ]

7. birth-order of the child In his/her family?....................

(A) Description of the Family members

Family member	Age	Educational Level	Job
Father	8.1	8.2	8.3
Mother	9.1	9.2	9.3
Brother 1	10.1	10.2	10.3
Brother 2	11.1	11.2	11.3
Sister 1	12.1	12.2	12.3
Sister 2	13.1	13.2	13.3

14. Mother's age at their marriage…………………….

15. Father's age at their marriage ……………………..

16. The birth order of his/ her father in his family ....

17. The birth-order of his/her mother in her family ....

18. The number of members in their family………

19. Does any family member has a long-standing abdominal pain? (more than two months?)

Yes ☐ No ☐

20.if the answer is the yes for 19 who is the family member with abdominal pain?

Mother ☐ Father ☐ Brother ☐ Sister ☐

21. Does any family member have a long-standing pain except abdominal pain?

(more than two months)

Yes ☐ No ☐

The antenatal history

22. Were there any complications during the pregnancy? Yes ☐ No ☐

23 Please mentioned any c**omplications occurred during the pregnancy**

Diabetes ☐ High blood pressure ☐ Bleeding ☐

Heart diseases ☐ growth failure of the baby ☐

Other complications………………………….

24 . Period of Gestation at the delivery of this child ……………………………….

25. Method of delivery of this child

1. Normal delivery ☐ 2. Emergency caesarian section ☐

3. Elective caesarian section ☐ 4. Forceps/vacuum delivery ☐

26 If caesarian delivery or forceps/vacuum delivery, Please explain the reason?

…………………………………………………………………………………………….

Post Partum period

27. How many days did the baby stayed at the hospital after the childbirth? ....

28. Did your child get any treatment at PBU/ Baby room ? ……………………….

29. If yes……………Please explain the reason?

30. Were there any complications developed by the baby soon after the delivery?

Yes ☐ No ☐

If the answer is yes please mentioned the disease

Change the skin colour(Yellow) ☐ infections ☐

Other complications ………………………………..

31. what was the duration of exclusive breastfeeding after childbirth

Four months ☐

Six months ☐

Other. ☐

Please mention the duration in months

32 Period of breastfeeding

Six months from birth ☐

Six months to one year ☐

1year -2 years ☐

More than two years ☐

Please mentioned the period of breastfeeding in months ……………………………

The records of medical history

33. The number of times child get medical care for abdominal pain during the **past one year**

……

34. The number of times child get medical care for abdominal pain during the **past three months**

35. No. of times child get medical advice except for abdominal pain during last year?

Medical advice	Doctor<br>1. Family doctor<br>2. Paediatrician<br>3. From hospital	Main symptoms	Result<br>1. Good more than previous<br>2. No change<br>3. Unsatisfied
37	33.1	33.2	33.3
38	34.1	34.2	34.3
39	35.1	35.2	35.3
40	36.1	36.2	36.3

The study of functional abdominal pain among 5-12 years old children

1. During the last two months Did your child have any abdominal pain?

   Yes ☐

   No ☐

2. If yes…..Please mark the site /sites with a cross/crosses(X) in the image given below

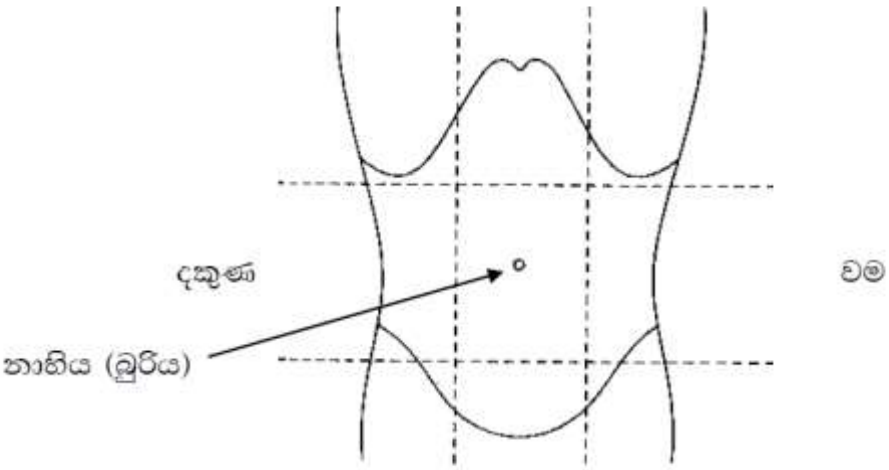

3. How long has the child been suffering from stomach ache?

4. During the last two months, how many times did your child get discomfort or pain?

Not at all	
1-3 times per month	
Once a week	
Several times per week	
Everyday	

5. Please mark the accompanied complaints with abdominal pain,

1. Pain in other body parts	yes		No	
2. Nausea	yes		No	
3. Abdominal bloating	yes		No	
4. Satiety with a small amount of food	yes		No	

6. The severity of pain or discomfort during the last two months

1 . mild	
11. moderate	
111 . some extent	
1V . Severe	

7. duration of Pain or discomfort one episode

1. Less than 1 hour ☐
11. 1-2 hours ☐
111. 3-4 hours ☐
1V. majority time of day ☐
V. Throughout the day ☐

8. How long has the child been suffering from pain or discomfort?

1 . Month or less ☐
11. 2 months ☐
111. 3 months ☐
1V. 4-11 months ☐
V. One Year or more ☐

Circle the answer.

During the last two months

9. How often has your child been cured of constipation or discomfort after defecation?

0 Never 1. Seldom 11. Sometimes 111. Most of the time 1V always

V. None

10. How often does your child have a frequent bowel movement or diarrhoea?

0 Never 1. Seldom 11. Sometimes 111. Most of the time 1V always

V. None

11. How often does your child have abdominal pain or discomfort with a hard stool than usual?

0 Never 1. Seldom 11. Sometimes 111. Most of the time 1V always

V. None

12. How often does your child have abdominal pain or discomfort with passing stools more times than other days?

0 Never 1. Seldom 11. Sometimes 111. Most of the time 1V always

V. None

13. How often does your child have abdominal pain or discomfort with passing stools less frequently?

0 Never 1. Seldom 11. Sometimes 111. Most of the time 1V always

V. None

14. How often does your child feel bloated with abdominal pain or discomfort?

0 Never 1. Seldom 11. Sometimes 111. Most of the time 1V always

V. None

15. How often does your child have abdominal pain or discomfort accompanied by headache.

0 Never 1. Seldom 11. Sometimes 111. Most of the time 1V always

V. None

16. How often does your child have abdominal pain or discomfort with sleeping interruption

0 Never 1. Seldom 11. Sometimes 111. Most of the time 1V always

V. None

17. How often does your child have abdominal pain or discomfort with body pain and backache

0 Never 1. Seldom 11. Sometimes 111. Most of the time 1V always

V. None

18. How often does your child have abdominal pain or discomfort accompanied by faintishness and vertigo.

0 Never 1. Seldom 11. Sometimes 111. Most of the time 1V always

V. None

19. How often does your child miss school due to stomach ache or discomfort?

0 Never 1. Seldom 11. Sometimes 111. Most of the time 1V always

V. None

20. Does your child get these Symptoms during the period of abdominal pain?

1. Loss of appetite	
2. Proximity to vomiting	
3. Vomiting.	
4. Paleness	
5. Headache	
6. Difficulty looking at light	

21. Does your child can do his day to day work during the period of abdominal pain?

Yes ☐ No ☐
