# Supplementary material for: Early life events in functional abdominal pain disorders in children
Source: PLoS One. 2022 Nov 2;17(11):e0275419. doi: 10.1371/journal.pone.0275419 (PMC9629606; doi:10.1371/journal.pone.0275419)
Supplement: Attachment — Submitted filename: PLOS One ver-3 -Response to Reviewers-30.07.2021.docx [file pone.0275419.s004.docx]

| Reviewer 1 comments | Response |
| --- | --- |
| Reviewer #1: Thank you for addressing the comments! | Your comments were very valuable, and we believe they made our manuscript more strong. |
| I have one more issue and I think that needs to be addressed. After looking the questionnaires I could see many technical terms used there in questionnaire like vertigo, elective cesarean section, emergency cesarean section and so on. Has the questionnaire been delivered in local language or English? | The questionnaire was developed in English and translated to the local language (Sinhalese). The questions were simple, and the technical terms were carefully translated so that a simple layman can understand the meaning. We pretested it in the hospital clinic and parents were able to understand the questionnaire without difficulty.  In addition, the telephone number of the first author was in the questionnaire and we encouraged the parents to call should they need clarifications regarding any question or a phrase. |
| As the questionnaire is self administered, how was this addressed? | Please see above. |
| If the local language translation were used, so far my knowledge it should be mentioned in the methods section. | We agree with the reviewer and a sentence was added into the method section stressing the language. |

PONE-D-20-15012R1

Early life events in functional abdominal pain disorders in children

PLOS ONE

Second revision

Responses for reviewers comments for version 2

| Reviewer 3 comments | Response |
| --- | --- |
| This is an interesting study on the association between certain early life events and functional abdominal pain disorders (FAPDs) in children. The following are my comments: | Thank you. |
| In the methods: Authors should clarify when the study was conducted. | The study was conducted in 2015. We included it into the manuscript. |
| • Methods; Selection of study participants: “Four such schools (that have classes from grades 1-7) were randomly chosen …... Two classes from each grade (1-7 years) were selected randomly…All children in the selected classes were invited to participate in the study”. How did the authors accurately include invited 1000 participants? Average number of students per class? | When we calculated sample size, the minimum number of children needed for the study came as 554. To make study data more robust we included 1000 participants. The average number of children in classes ranged from 20-35. |
| • Methods; Data collection: “Parents were invited to fill all the questionnaires and the consent forms, seal them in the envelope provided, and return them to school”. Non-response may be attributed to parents being illiterate (selection bias)? | The literacy rate in Sri Lanka is >95% and it is extremely unlikely that both parents are illiterate in a given home. The questionnaire was in the local language, and we developed it very carefully that an average person could understand it without any difficulty. It was noted that the questionnaire could be understood by an average person without a difficulty during pretesting. |
| • Methods; Data analysis: Authors have to clarify all independent variables analyzed in their study, including composite variables (e.g., pre-natal complications, post-natal complications) and used cutoffs (e.g., parental education; ordinary level?). Being the main focus in this study, pre-natal and post-natal complications should also be individually reported. | In the revised manuscript key pre and postnatal events were individually reported (table 1) |
| The authors didn’t report whether there are statistically significant differences between children with and without FABDs in some important characteristics, such as gender and residence. | Table 1 depicts all demographic variables and in that table, most of the sociodemographic factors of the cases and controls were compared. The study was conducted in a semi-urban setting and therefore residence was not included in the comparison as there would not be a difference in this variable. |
| • Authors investigated the association of several independent variables with FAPDs, and found some statistically significant relations using bivariable analysis. However, multivariable statistical analysis (e.g., logistic regression) is required to control possible confounding factors. | In the revised manuscript, Table 2 depicts the binary logistics regression to determine the early life events as the predictors of development of FAPDs |
| Moreover, authors should explain in the discussion the possibility of other confounding factors that are not included in this study, such as psychological factors, surgeries, and infections. | This valuable suggestion was taken into consideration and a sentence was included into the limitations |
| • In the discussion: “we noted a significant association between admission to SCBUs and the development of FAPDs”. However, the authors didn’t report this in the results section. | This variable is in table 1. |
| • The study design can allow determining associations but not causal-relationships. That is, authors should revise wordings, such as: In the abstract: “The extended period of breastfeeding (>2years) was shown to reduce the occurrence of FAPDs”. | We agree with the reviewer. The wording implying casual-relationship was changed to associations |
| • Authors should acknowledge, in the manuscript, their use of ROME III criteria, rather than the most updated ROME IV criteria, as one of the study limitations. | When we conducted the study in 2015 the available version of Rome criteria was Rome III. Therefore, we do not see it as a limitation. |
