# Supplementary material for: Early life events in functional abdominal pain disorders in children
Source: PLoS One. 2022 Nov 2;17(11):e0275419. doi: 10.1371/journal.pone.0275419 (PMC9629606; doi:10.1371/journal.pone.0275419)
Supplement: Attachment — Submitted filename: PLOS one -third-revision-response to reviewers-29.11.2021.docx [file pone.0275419.s005.docx]

PONE-D-20-15012R2

Early life events in functional abdominal pain disorders in children

PLOS ONE

| **Comments to the Author**  1. If the authors have adequately addressed your comments raised in a previous round of review and you feel that this manuscript is now acceptable for publication, you may indicate that here to bypass the “Comments to the Author” section, enter your conflict of interest statement in the “Confidential to Editor” section, and submit your "Accept" recommendation. | **Response** |
| --- | --- |
| Reviewer #1: All comments have been addressed | Thank you |
| Reviewer #3: (No Response) |  |
|  |  |
| 2. Is the manuscript technically sound, and do the data support the conclusions?  The manuscript must describe a technically sound piece of scientific research with data that supports the conclusions. Experiments must have been conducted rigorously, with appropriate controls, replication, and sample sizes. The conclusions must be drawn appropriately based on the data presented. |  |
| Reviewer #1: Yes | Thank you |
| Reviewer #3: No | All the suggestion made by reviewers in the last revision was adequately addressed |
|  |  |
| 3. Has the statistical analysis been performed appropriately and rigorously? |  |
| Reviewer #1: Yes | Thank you |
| Reviewer #3: No | All the suggestion made by reviewers in the last revision was adequately addressed |
|  |  |
| 4. Have the authors made all data underlying the findings in their manuscript fully available?  The [PLOS Data policy](http://www.plosone.org/static/policies.action#sharing) requires authors to make all data underlying the findings described in their manuscript fully available without restriction, with rare exception (please refer to the Data Availability Statement in the manuscript PDF file). The data should be provided as part of the manuscript or its supporting information, or deposited to a public repository. For example, in addition to summary statistics, the data points behind means, medians and variance measures should be available. If there are restrictions on publicly sharing data—e.g. participant privacy or use of data from a third party—those must be specified. |  |
| Reviewer #1: Yes |  |
| Reviewer #3: Yes |  |
|  |  |
| 5. Is the manuscript presented in an intelligible fashion and written in standard English?  PLOS ONE does not copyedit accepted manuscripts, so the language in submitted articles must be clear, correct, and unambiguous. Any typographical or grammatical errors should be corrected at revision, so please note any specific errors here. |  |
| Reviewer #1: Yes |  |
| Reviewer #3: Yes |  |
|  |  |
| 6. Review Comments to the Author  Please use the space provided to explain your answers to the questions above. You may also include additional comments for the author, including concerns about dual publication, research ethics, or publication ethics. (Please upload your review as an attachment if it exceeds 20,000 characters) |  |
| Reviewer #1: The comments have been addressed well by the authors. So far on my knowledge, the manuscript has been well organized with the details than earlier version submitted. | Thank you |
| Reviewer #3: The authors successfully addressed most reviewers' comments. However, authors should clarify statistical methods used in Table 2, why these variables were specifically included, and what are the interpretation of results? | There was a suggestion in last review as follows  Quote  *Authors investigated the association of several independent variables with FAPDs, and found some statistically significant relations using bivariable analysis. However, multivariable statistical analysis (e.g., logistic regression) is required to control possible confounding factors.*  Unquote  Therefore , regression analysis was done and adequately discussed in the discussion |
|  |  |
| 7. PLOS authors have the option to publish the peer review history of their article ([what does this mean?](https://journals.plos.org/plosone/s/editorial-and-peer-review-process#loc-peer-review-history)). If published, this will include your full peer review and any attached files.   If you choose “no”, your identity will remain anonymous but your review may still be made public.   **Do you want your identity to be public for this peer review?** For information about this choice, including consent withdrawal, please see our [Privacy Policy](https://www.plos.org/privacy-policy). |  |
| Reviewer #1: No |  |
| Reviewer #3: **Yes:** Elsayed Abdelkreem |  |

| Authors investigated the association of several independent variables with FAPDs, and found some statistically significant relations using bivariable analysis. However, multivariable statistical analysis (e.g., logistic regression) is required to control possible confounding factors. | In the revised manuscript, Table 2 depicts the binary logistics regression to determine |
| --- | --- |

|  | the early life events as the predictors of development of FAPDs |
| --- | --- |
