# Supplementary material for: Early life events in functional abdominal pain disorders in children
Source: PLoS One. 2022 Nov 2;17(11):e0275419. doi: 10.1371/journal.pone.0275419 (PMC9629606; doi:10.1371/journal.pone.0275419)
Supplement: Attachment — Submitted filename: Response to Reviewers-ver-5-24.3.2022.docx [file pone.0275419.s006.docx]

PONE-D-20-15012R3

Early life events in functional abdominal pain disorders in children

PLOS ONE

| **Comments to the Author**  1. If the authors have adequately addressed your comments raised in a previous round of review and you feel that this manuscript is now acceptable for publication, you may indicate that here to bypass the “Comments to the Author” section, enter your conflict of interest statement in the “Confidential to Editor” section, and submit your "Accept" recommendation. | **Authors response** |
| --- | --- |
| Reviewer #3: (No Response) |  |
|  |  |
| 2. Is the manuscript technically sound, and do the data support the conclusions?  The manuscript must describe a technically sound piece of scientific research with data that supports the conclusions. Experiments must have been conducted rigorously, with appropriate controls, replication, and sample sizes. The conclusions must be drawn appropriately based on the data presented. |  |
| Reviewer #3: Partly | All the revisions proposed by the reviewers were addressed appropriately to generate a technically sound manuscript |
|  |  |
| 3. Has the statistical analysis been performed appropriately and rigorously? |  |
| Reviewer #3: No | The statistical analysis was improved in the revised manuscript which include the adjusted Odds Ratio (AOR) in the multivariate analysis model. |
|  |  |
| 4. Have the authors made all data underlying the findings in their manuscript fully available?  The [PLOS Data policy](http://www.plosone.org/static/policies.action#sharing) requires authors to make all data underlying the findings described in their manuscript fully available without restriction, with rare exception (please refer to the Data Availability Statement in the manuscript PDF file). The data should be provided as part of the manuscript or its supporting information, or deposited to a public repository. For example, in addition to summary statistics, the data points behind means, medians and variance measures should be available. If there are restrictions on publicly sharing data—e.g. participant privacy or use of data from a third party—those must be specified. |  |
| Reviewer #3: Yes | Thank you |
|  |  |
| 5. Is the manuscript presented in an intelligible fashion and written in standard English?  PLOS ONE does not copyedit accepted manuscripts, so the language in submitted articles must be clear, correct, and unambiguous. Any typographical or grammatical errors should be corrected at revision, so please note any specific errors here. |  |
| Reviewer #3: Yes | Thank you |
|  |  |
| 6. Review Comments to the Author  Please use the space provided to explain your answers to the questions above. You may also include additional comments for the author, including concerns about dual publication, research ethics, or publication ethics. (Please upload your review as an attachment if it exceeds 20,000 characters) |  |
| Reviewer #3: The authors didn't address prior comments related to clarification and justification of Table 2 and used multivariate analysis. In the statistical analysis, authors stated "We used binary logistic regression to determine the independent association between early life events and FAPDs which were shown to be significant in the univariate analysis.". However, authors should define the cut-off for p value used to select the included items as well as adjust for other variables that showed significant statistical difference in the univariate analysis. In the multivariate analysis model, authors should report adjusted Odds Ratio (AOR), rather than just OR, in order to investigate the independent factors. | The statistical analysis was improved in the revised manuscript which include the adjusted Odds Ratio (AOR) in the multivariate analysis model. |
|  |  |
| 7. PLOS authors have the option to publish the peer review history of their article ([what does this mean?](https://journals.plos.org/plosone/s/editorial-and-peer-review-process#loc-peer-review-history)). If published, this will include your full peer review and any attached files.   If you choose “no”, your identity will remain anonymous but your review may still be made public.   **Do you want your identity to be public for this peer review?** For information about this choice, including consent withdrawal, please see our [Privacy Policy](https://www.plos.org/privacy-policy). |  |
| Reviewer #3: **Yes:** Elsayed Abdelkreem | Your suggestions for improvements are highly appreciated |
|  |  |
