# Supplementary material for: Early life events in functional abdominal pain disorders in children
Source: PLoS One. 2022 Nov 2;17(11):e0275419. doi: 10.1371/journal.pone.0275419 (PMC9629606; doi:10.1371/journal.pone.0275419)
Supplement: Attachment — Submitted filename: ELE-PLOS ONE-Reviewer comments-19.12.2020.docx [file pone.0275419.s003.docx]

Reviewer comments

**Reviewer #1:**

Comment

First of all, congratulations to the authors for the interesting paper. The findings of the study are really remarkable and the outcomes would be of much value in the pediatric patients for the prevention of Functional Abdominal Pain Disorders.

Response

Thank you

There are few comments from my side on the paper which I think should be made clear for the further validity and reliability of the paper before publishing.

Comment
1. My first comment is on the criteria used for the diagnosis of FAPD. There has been recent update on the ROME III criteria to ROME IV criteria with some significant changes. Is there any rationale for the use of ROME III criteria and not ROME IV criteria in the study?

Response:

We conducted this study in 2015. During that time Rome IV criteria were not available. Therefore, we used Rome III criteria which were the latest at that time. When the newly generated Rome IV criteria were released in 2016, there was a major change in definitions of all abdominal pain related functional gastrointestinal disorders. That was the reduction of duration of symptoms from two months to one month. Therefore, we were unable to use Rome IV criteria

Comment
2. My second comment is on the methodology regarding sampling methods followed in the study. How was the random selection of classes and students done?Also is the sample size adequate? Is there any basis or rationale for the given sample size in the study?

Response:

Details about sample calculation was included into the manuscript. The necessary sample size was 554. To make our data more robust we included 1000 children. Random selection of schools and classes were done by using a random number generator. (www.random.org)

**Sample size and sampling technique**

The sample size for this study was calculated by using the following formula (Lowanga, Lameshow 1991).

Size or the sample	N = Z2 P (1-P) /d^2^

Z = 1.96

P = anticipated population proportion

D = absolute precision required for the estimate to fall within a given percentage point of the proportion = 2.5%.

10% was taken as the value for P from previous Sri Lankan study (Devanarayana, Mettananda et al. 2011).

P=10%

N = Z^2^ P (1-P) /d^2^

=1.96*1.96*0.9/0.025*0.025= 3.45744/0.00625 = 553.19

Substituting the above values in the equation the required sample size proved to be 553. The response rate was anticipated as 60%. Therefore, to achieve the desired sample size, it was decided to recruit 1000 school children.

Comment
3. The questionnaire used for data collection is not provided as supplementary files. Could you please provide the validated questionnaire used in the study?

Response

We included the questionnaires used in this study as supplementary materials

Comment
4. Is there any basis for significant relationship of parental factors like education level and jobs related to the FAPD in the children? They are found to be statistically significant in the study but not discussed in discussion section.

Response

In the revised manuscript, we discussed these factors and their relevance.

Comment
5. There are many typographical errors in the manuscript. For example, in results section sample characteristics sub heading the following second sentence is not clear.
"The control group consisted of 571 children. There were 292 (51.1%) [mean age of 8.85 years (SD 2.0 years)] in the control group."
Font of the text is also not uniform in the manuscript and there are grammatical errors.

Response

We corrected all typographic and grammatical errors in the revised version.

**Reviewer #2:**

 Introduction:

Comment:
In paragraph 3: authors tried to relate or see commonalities between gastric suction and adverse prenatal and post-natal complications that would predispose children to develop FAPDs…..this need to be explained how?

Response

Paragraph 3 was changed. We included more detailed explanation regarding neonatal pain related events and the logical reasons for conduct the study.

Comment
In paragraph 4: psychological interventions are promising but challenging to apply in a clinical setting…..which psychological interventions are being referred here? If would be nice if this can get properly referred?

Response

We included details about psychological interventions of childhood FAPDs and appropriate references to the paragraph.

Comment
Therefore, understanding of ELEs that predispose children to FAPDs would
give us a window of opportunity to prevent them from developing similar diseases……should be replaced by reducing the incidence of similar disease.

Response:

Corrected as suggested by the reviewer

Methods:

Comment
Four mixed schools that had classes from grade 7 or above were selected randomly from those schools by drawing lots…….not understood fully what “drawing lots” means?

Response

Details about the random selection of schools and classes were included into the manuscript.

Comment
“Two classes from each grade (grade 1-7) were selected randomly from those selected schools”…..just a sentence before it’s being said grade 7 and above? This should be corrected by clearly stating is it grade 7 and above or between grade 1-7.

Response
The section was re-written with more details and the error was corrected. The grades were grades 1 to 7 not grade 7 or above. We would like to thank the reviewer for pointing out this mistake.

Data collection:

Comment
The following information seems redundant:
“Children handed over the questionnaires to the class teacher who collected them. Investigators collected all returned questionnaires from the class teachers. We sent two reminders to parents who did not return the completed questionnaires”

Response:

The sentences were edited.

Results:
Comment
“A thousand questionnaires were distributed”……I think it should be replaced by One thousand questionnaires were distributed.

Response

Corrected as suggested by the reviewer.

Comment

“The control group consisted of 571 children. There were 292 (51.1%) [mean age of 8.85 years (SD 2.0 years)] in the control group” ……..Either there is a mistake here or I am confused??? Is the number for control group 571 or 292 children???

Response

We thank the reviewer for pointing out this mistake. It was corrected.

It should have been “The control group consisted of 571 children. There were 292 males (51.1%)”

Comment

“There were 86 children with FAP (47.3%), 48 with IBS (26.4%), 31 with FD (17.0%) and 11
with AM (6%). Six (3.2 %) children had more than one AP-FGIDs”……should it be FAPDs

Response

Corrected as suggested by the reviewer

Discussion:

Comment

On Page 19 line 1 “In addition to these factors, we also identified the mode of delivery other than normal
vaginal delivery was also identified as a probable risk factor for developing” FAPDs……..highlighted “was also identified” should be deleted from this sentence.

Response

Corrected as suggested by the reviewer

Comment
On page 20, in 2nd paragraph, “Receiving intravenous antibiotics in early neonatal period is likely to interfere with the development of the gut microbiota and the brain-gut-microbiota axis of the newborn”. ……please replace with “Receiving intravenous antibiotics in early neonatal period is likely to interfere with the development and possible alteration of the gut microbiota and the brain-gut-microbiota axis of the newborn”.

Response

Corrected as suggested by the reviewer
